# Multi-Functional Roles of Chitosan as a Potential Protective Agent against Obesity

**DOI:** 10.1371/journal.pone.0053828

**Published:** 2013-01-14

**Authors:** Ann M. Walsh, Torres Sweeney, Bojlul Bahar, John V. O’Doherty

**Affiliations:** 1 School of Agriculture and Food Science, University College Dublin, Lyons Research Farm, Newcastle, Co. Dublin, Ireland; 2 School of Veterinary Medicine, University College Dublin, Belfield, Co. Dublin, Ireland; Virginia Tech, United States of America

## Abstract

Chitosan, a natural polysaccharide comprising copolymers of glucosamine and N-acetylglucosamine, has been shown to have anti-obesity properties. Two experiments (Exp. 1 and Exp. 2) were performed to determine the role of chitosan on dietary intake, body weight gain, and fat deposition in a pig model, as well as identifying potential mechanisms underlying the anti-obesity effect of chitosan. In Exp. 1, the nutrient digestibility experiment, 16 pigs (n = 4/treatment) were randomly allocated to one of four dietary treatments as follows: 1) basal diet; 2) basal diet plus 300 ppm chitosan; 3) basal diet plus 600 ppm chitosan; 4) basal diet plus 1200 ppm chitosan. The main observation was that crude fat digestibility was lower in the 1200 ppm chitosan group when compared with the control group (*P*<0.05). In Exp. 2, a total of 80 pigs (n = 20/treatment) were offered identical dietary treatments to that offered to animals in Exp. 1. Blood samples were collected on day 0, day 35 and at the end of the experiment (day 57). Animals offered diets containing 1200 ppm chitosan had a lower daily dietary intake (*P*<0.001) and body weight gain (*P*<0.001) from day 35 to 57 when compared with all the other treatment groups. Animals offered diets containing 1200 ppm chitosan had a significantly lower final body weight (*P*<0.01) when compared with all the other treatment groups. The decreased dietary intake observed in the 1200 ppm chitosan group was associated with increased serum leptin concentrations (*P*<0.001) and a decrease in serum C-reactive protein (CRP) concentrations (*P*<0.05). In conclusion, the results of this study highlight novel endocrine mechanisms involving the modulation of serum leptin and CRP concentrations by which chitosan exhibits anti-obesity properties *in vivo*.

## Introduction

Obesity is recognized as a chronic disease characterised by the overaccumulation of fat stores in adipocytes and is frequently linked with type 2 diabetes, inflammation, hypertension and cardiovascular diseases [1], [2]. The potential of natural products to prevent obesity have been highlighted [3], with evidence to suggest that chitosan has anti-obesity effects [4], [5]. Chitosan is a polysaccharide comprising copolymers of glucosamine (β(1–4)-linked 2-amino-2-deoxy-_D_-glucose) and N-acetylglucosamine (2-acetamido-2-deoxy-_D_-glucose) and can be derived by partial deacetylation of chitin [6]. Chitosan is a non-toxic nutritional supplement generally regarded as a safe compound [7], [8].

It has been generally accepted that the anti-obesity effects of chitosan originate from its unique fat-binding properties, which interferes with the absorption of dietary lipids from the gastrointestinal tract [9], [10]. However, more recent research has suggested a more complex mode of action for chitosan: a decrease in feed intake was recorded in mice supplemented with chitosan [1], [11], while exposure of pre-adipocytes to chitooligosaccharide modulated adipokine secretion and inhibited adipogenesis *in vitro* [12]. Adipose tissue is an active participant in controlling the body’s patho-phsiological processes by releasing a variety of adipokines into the blood stream [1]. These adipokines include leptin, tumour necrosis factor alpha, interleukin-6 (IL-6) and C-reactive protein (CRP) [1], [13]. These adipokines, through their local and systemic actions, are postulated to regulate energy metabolism, inflammation and insulin sensitivity [11], [14]. The potential anti-obesity effects of chitosan *in vivo* may include the modulation of adipokines, suggesting a second possible endocrine mechanism of action of chitosan. Hence, the present study was undertaken to assess the potential of chitosan supplementation as an anti-obesity functional food by using the pig as a model. The first aim of this study was to investigate the effect of varying levels of dietary chitosan on body weight gain, diet intake, nutrient digestibility, selected microflora populations and carcass characteristics. The second aim was to determine if dietary chitosan could modify adipokines such as leptin, IL-6 and CRP *in vivo*.

## Materials and Methods

### Ethical Approval

All procedures described in this experiment were conducted under experimental licence from the Irish Department of Health in accordance with the Cruelty to Animals Act 1876 and the European Communities (Amendments of the Cruelty to Animals Act 1876) Regulations, 1994. An experimental license (AREC-09-31-O’Doherty) was obtained from the Animal Research Ethics Subcommittee University College Dublin.

### Experimental Design and Diets

Two experiments (Exp. 1 and Exp. 2) were performed by using a complete randomised design with four treatment groups to evaluate the response of animals to varying levels of dietary chitosan. The dietary treatments were as follows: 1) basal diet (0 ppm chitosan); 2) basal diet plus 300 ppm chitosan; 3) basal diet plus 600 ppm chitosan; 4) basal diet plus 1200 ppm chitosan. The range of chitosan concentrations used were based on previous work by Bahar et al [12] and O’Shea et al [15]. All diets were formulated to have identical concentrations of digestible energy (13.9 MJ/kg) and standardised ileal digestible lysine (8.5 g/kg). All other amino acid requirements were met relative to lysine according to the ideal protein concept [16]. The chitosan was sourced from A&Z Food Additives Co. Ltd (Hangzhou, China) and contained 90.2% deacetylation. All diets were offered in meal form. The dietary composition and analysis are presented in Table 1.

**Table 1:** Composition and chemical analysis of basal diet^1^ (g/kg, unless otherwise indicated).

Composition	Basal diet
Wheat	382.6
Barley	250.0
Soya bean meal	170.0
Maize	150.0
Soya oil	18.0
Limestone	12.5
Salt	5.0
Monocalcium phosphate	6.6
Vitamins and minerals premix^2^	2.5
Lysine HCl	2.3
L-threonine	0.5
**Analysis**	
Dry matter	895.9
Crude protein (N X 6.25)	177.9
Neutral detergent fibre	130.5
Ash	43.5
Gross energy (MJ/kg)	16.1
Lysine^3^	9.2
Methionine and cysteine^3^	5.5
Threonine^3^	6.2
Tryptophan^3^	1.9
Calcium^3^	9.4
Phosphorous^3^	5.8

1 Treatments: 1) basal diet; 2) basal diet plus 300 ppm chitosan; 3) basal plus 600 ppm chitosan; 4) basal plus 1200 ppm chitosan.

2 The premix provided vitamins and minerals (per kg diet) as follows: 4.2 mg of retinol, 0.07 mg of cholecalciferol, 80 mg of α-tocopherol, 120 mg of cupricas cuprous sulphate, 100 mg iron as ferrous sulphate, 100 mg of zinc as zinc oxide, 0.3 mg of selenium as sodium selenite, 25 mg of manganese as manganous oxide, 0.2 mg of iodine as calcium iodate on a calcium sulphate/calcium carbonate carrier, 2 mg of thiamine, 15 µm of cyanocobalamin, 7 mg of pantothenic acid, 2 mg of riboflavin, 7 mg of niacin, 3 mg of adenine and 100 mg of phytase (Natuphos) (Nutec, Co. Kildare, Ireland).

3 Calculated for tabulated nutritional composition [48].

### Experiment 1: Nutrient Digestibility

Sixteen finishing boars (progeny of meat line boars x (Large White x Landrace sow)) with an initial body weight of 75 (SD = 2.10) kg bodyweight were used in this nutrient digestibility study. The animals were blocked on the basis of live weight and litter of origin and were randomly allocated to one of four dietary treatments. The animals were allowed a 14 day dietary adaptation period, weighed and transferred to individual metabolism crates that facilitated total but separate collections of urine and faeces. Entire boars were used in this experiment for ease of collecting urine and faeces separately. The animals were given a further 6 days to acclimatise to the metabolism crates before collections begun for a further 7 days to determine apparent digestibility of nutrients. The animals were fed *ad libitum* (measured during the adaptation period). Fresh water was also provided *ad libitum*. The metabolism crates were located in a temperature-controlled room, maintained at 22°C. During collections, urine was collected in a plastic container below the crate. Total faeces weight was recorded daily and oven dried at 100°C. At the end of the collection period, the faeces samples were pooled and a sub-sample retained for laboratory analysis. Feed samples were collected each day and retained for chemical analysis.

### Experiment 2: Dietary Intake and Endocrine Mechanism

Eighty finishing gilts of 65 (SD = 2.5) kg bodyweight were used in this experiment, which was run in parallel with Exp. 1, from same genetic stock. The animals were blocked on the basis of live weight and litter of origin and were randomly assigned to one of the four dietary treatments used in the Exp. 1. Animals were penned in groups of ten with an individual space allowance of 0.75 m^2^. The experiment was run over two time periods with ten pigs of each treatment examined per time period. The house was mechanically ventilated to provide an ambient temperature of 18°C. Each pen had a solid floor lying area with access to slats at rear. The four group-pens were equipped with single-space computerized feeders (Mastleistungsprufung MLP-RAP; Schauer Agrotronic AG, Sursee, Switzerland). When the animal entered the feeder, it was recognized by the electronic system (MLP-Manager 1.2; Schauer Maschinenfabrik Ges.m.b.H and CoKG, Prambachkirchen, Austria). Each animal was fitted with a uniquely coded ear tag transponder and the identification circuit recorded the animal’s number. When the animal finished feeding and withdrew from the trough, the electronic system recorded the difference between the pre- and post-visit trough weight and the data were stored in a file with the pen number, the animal’s identification number, and the date and the time of entry and exit as described by Varley *et al.* [17]. The recorded data were used to calculate the individual dietary intake. The body weight of each animal was measured at the start of the experiment and subsequently on day 17, day 35 and day 57, and dietary intake was monitored daily.

### Blood Collection

Blood samples (10 ml) were taken from 5 animals per treatment per run (total of 10 per treatment) and were collected from the *vena jugularis* by puncture into vacutainers (Becton, Dickinson, Drogheda, Ireland) on day 0 (prior to commencing of the experiment), day 35 and day 57 to facilitate adipokine (leptin, IL-6 and CRP) quantification. Blood samples were collected from the same pigs at each time point. Blood samples were allowed to clot at 4°C, and serum was collected after centrifugation (1,500×g for 15 min at 4°C). Serum samples were stored at −20°C until analysis.

### Carcass Analysis

All the animals were sacrificed on day 57 of the experiment. Backfat thickness was measured at 6 cm from the edge of the split back at the level of the third and fourth last rib by using the Hennessy grading probe (Hennessy and Chong, Auckland, New Zealand). The lean meat content was estimated according to the following formula [18]:

Estimate lean meat content (g/kg) = 543.1–7.86x +2.66y.

Where x is fat depth (mm) and y is muscle depth (mm).

Further carcass data were determined by application of the following equations:

Carcass weight (kg) = Hot carcass weight x 0.98.

Kill-out proportion (%) = Carcass weight/body weight.

Carcass ADG (kg/day) = Carcass weight – (initial body weight x 0.65)/number of days on.

experiment.

Estimated ash content = 3% of carcass weight [19].

Carcass fat content = Carcass weight – (lean+ash content of carcass).

### Microbiology Study

Selected intestinal bacterial populations were measured as research has shown that a weight loss intervention based on a decrease in energy intake has an important impact on the composition of the gut microbiota of overweight individuals with *Lactobacillus* populations been one the most amendable gut bacteria to dietary intervention [20]. As a large number of intestinal bacterial species are unculturable [21], lactobacilli were enumerated as a reflection of changes in the population structure of beneficial bacteria. The relevance of measuring *Enterobacteriaceae* populations as an indicator of pathogenic bacteria is debated; however, intestinal inflammation has been related to a marked increase in *Enterobacteriaceae* numbers [22], [23]. Following sacrifice, digesta samples (approximately 10 g ±1 g) were aseptically recovered from the proximal colon in sterile conditions. Digesta samples were stored in sterile containers (Sarstedt, Wexford, Ireland), placed in insulated containers with dry ice, and transported to the laboratory within 3 h. Populations of lactobacilli and *Enterobacteriaceae* were selectively isolated and enumerated. Lactobacilli were isolated on agar (de Man, Rogasa, Sharpe agar; Oxoid Ltd., Hampshire UK) with overnight (18 to 24 h) incubation at 37°C in a 5% CO_2_ environment. A carbohydrate fermentation-based identification kit (API 50 CHL, BioMerieux, Marcy l’Etoile, France) was used to confirm suspect lactobacilli. *Enterobacteriaceae*, including *Escherichia coli,* were isolated on an agar (MacConkey, Oxoid Ltd., Hampshire, UK), after aerobic incubation at 37°C overnight (18 to 24 h). Suspect colonies were confirmed (API 20E system, BioMerieux, Marcy l’Etoile, France). Typical colonies of each bacterium were counted, log transformed and presented per gram of digesta.

### Laboratory Analysis of Samples

The dry matter (DM) of the diets and faeces were determined after drying for 24 h at 100°C. The crude ash content of diets and faeces was determined after ignition of a weighed sample in a muffle furnace (Nabertherm, Bremen, Germany) at 500°C for 6 h. The gross energy (GE) of diets and faeces samples was determined by using an adiabatic bomb calorimeter (Parr Instruments, IL, USA). The neutral detergent fibre (NDF) fraction of diets and faeces was analysed by using a Fibertec extraction unit (Tecator, Hoganans, Sweden) [24]. The nitrogen (N) concentration of diets and faeces was determined by using a LECO FP 528 instrument (Leco Instruments, U.K. Ltd, Newby Road, Hazel Grove, Stockport, SK7 5DA, Cheshire). The oil content was determined by using the Ether Extract Method B [25]. Serum leptin was quantified by using a specific pig leptin enzyme-linked immunosorbent assay (ELISA) kit from Uscn Life Science Inc. (Wuhan, China) according to the manufacturer’s instructions. Sensitivity of the assay was 0.114 ng/ml, and intra-assay coefficient of variation was <12%. Serum IL-6 was measured by using the porcine IL-6 duo set ELISA development system (R&D Systems Europe, Limited, Abingdon, UK), following the manufacturer’s instructions. Serum CRP was measured by using the porcine CRP duo set ELISA development system (R&D Systems Europe, Limited, Abingdon, UK), following the manufacturer’s instruction. Absorbance was measured at 450 nm against 570 nm for each assay by using the ELISA plate reader. All of the samples were assayed in duplicate in the same assay.

### Statistical Analysis

Data from both experiments were analysed by using the General Linear Model (GLM) procedure of Statistical Analysis System [26]. Data of body weight gain, dietary intake, nutrient digestibility, selected microbial populations and carcass data were analysed as a completely randomised design, with the animal as the experimental unit. The statistical model used included effect of dietary treatment, run and interaction between treatment and run. For serum leptin and CRP analysis, the data were analysed by repeated measures analysis using the Proc Mix procedure of SAS [27]. The model included the effects of treatment and time and the associated interaction. Multiple regression models were used to determine the relationship between leptin and dietary intake. Probability values of less than 0.05 were used as the criterion for statistical significance. All results are presented as least square means ± SEM.

## Results

### Nutrient Digestibility

The effects of varying chitosan inclusion level on nutrient digestibility are shown in Table 2. The crude fat digestibility was lower in the 1200 ppm chitosan group when compared with the control (*P*<0.05). However, inclusion of chitosan in the diet had no significant effect on DM intake, urine output, DM digestibility, organic matter (OM) digestibility, NDF digestibility, GE digestibility or ash digestibility (*P*>0.05).

**Table 2:** Effects of varying chitosan inclusion level on nutrient digestibility.

	Dietary chitosan, ppm					
	0	300	600	1200	SEM	*P*-value
**Intake & excretory parameters**						
Dry matter intake (kg/day)	2.294	2.334	2.133	2.252	0.0914	0.516
Urine output (kg/day)	5.535	5.062	4.746	4.998	0.6979	0.874
**Digestibility coefficients**						
Dry matter	0.875	0.872	0.877	0.875	0.0061	0.979
Organic matter	0.887	0.884	0.890	0.886	0.0068	0.961
Neutral detergent fibre	0.740	0.740	0.744	0.759	0.0161	0.778
Gross energy	0.862	0.857	0.866	0.860	0.0086	0.929
Ash	0.664	0.640	0.615	0.660	0.0247	0.675
Crude fat	0.788^b^	0.722^ab^	0.742^ab^	0.704^a^	0.0300	0.049

Values represents least square means (n = 4).

Means with the same superscript within rows are not significantly different (P>0.05).

### Dietary Intake and Body Weight

The effects of varying chitosan inclusion level on dietary intake, body weight gain and feed efficiency ratio of the animals are shown in Table 3. Dietary supplementation with chitosan had no effect on daily dietary intake, daily body weight gain or feed efficiency from day 0 to 35 of the experiment (*P*>0.05). However, animals offered diets containing 1200 ppm chitosan had a lower daily dietary intake (*P*<0.001), body weight gain (*P*<0.001), and feed efficiency ratio (*P*<0.01) from day 35 to 57 when compared with all the other treatment groups. Animals offered diets containing 1200 ppm chitosan had a lower daily body weight gain (*P*<0.01) during the entire experimental period (day 0 to 57) and lower final body weight (*P*<0.01) when compared with all the other treatment groups. Animals offered diets supplemented with 300 ppm, 600 ppm and 1200 ppm chitosan had a lower overall feed efficiency ratio (day 0 to 57) compared with the control treatment group (*P*<0.01).

**Table 3:** Effect of varying chitosan inclusion level on dietary intake, body weight gain and feed efficiency ratio of the animals.

	Dietary chitosan, ppm					
	0	300	600	1200	SEM	*P*-value
Final body weight (kg)	116.95^b^	115.99^b^	114.80^b^	109.89^a^	1.377	0.003
**Day 0 to 17**						
Daily dietary intake(kg/day)	2.295	2.128	2.226	2.227	0.0671	0.387
Daily chitosanintake (g/day)	0.000^a^	0.640^b^	1.326^c^	2.675^d^	0.0470	<0.0001
Daily body weightgain (kg/day)	0.998	0.960	0.926	0.937	0.0403	0.621
Feed efficiencyratio^1^ (kg/kg)	0.442	0.476	0.412	0.430	0.0193	0.128
**Day 17 to 35**						
Daily dietary intake(kg/day)	2.641	2.730	2.701	2.695	0.0722	0.858
Daily chitosanintake (g/day)	0.000^a^	0.820^b^	1.615^c^	3.237^d^	0.0633	<0.0001
Daily body weightgain (kg/day)	0.991	0.976	0.921	0.920	0.0470	0.613
Feed efficiencyratio (kg/kg)	0.375	0.360	0.341	0.341	0.0158	0.369
**Day 35 to 57**						
Daily dietary intake(kg/day)	2.908^b^	3.042^b^	2.945^b^	2.389^a^	0.0848	<0.0001
Daily chitosanintake (g/day)	0.000^a^	0.913^b^	1.762^c^	2.870^d^	0.0708	<0.0001
Daily body weightgain (kg/day)	0.962^b^	0.954^b^	0.940^b^	0.654^a^	0.0460	<0.0001
Feedefficiency ratio(kg/kg)	0.332^b^	0.317^b^	0.317^b^	0.268^a^	0.0131	0.005
**Day 0 to 57**						
Daily dietary intake(kg/day)	2.379	2.623	2.435	2.445	0.0680	0.071
Daily chitosanintake (g/day)	0.000^a^	0.788^b^	1.455^c^	2.936^d^	0.0510	<0.0001
Daily body weightgain (kg/day)	0.977^b^	0.965^b^	0.943^b^	0.861^a^	0.0259	0.009
Feed efficiency ratio(kg/kg)	0.424^b^	0.372^a^	0.384^a^	0.360^a^	0.0120	0.003

Values represents least square means (n = 20).

Means with the same superscript within rows are not significantly different (P>0.05).

1 Feed efficiency ratio; body weight gain (kg)/dietary intake (kg).

### Carcass Characteristics

The effects of varying chitosan inclusion level on carcass characteristics are shown in Table 4. Animals offered diets containing 1200 ppm had a significantly lower carcass weight (*P*<0.01) and carcass average daily gain (*P*<0.01) when compared with all the other treatment groups. Animals offered diets containing 1200 ppm had a significantly lower estimated carcass fat content (*P*<0.05) compared with the control and 600 ppm chitosan treatments. The supplementation of varying levels of chitosan had no significant effect on kill out percentage, back fat depth, lean percentage, lean yield and muscle depth (*P*>0.05).

**Table 4:** Effects of varying chitosan inclusion level on carcass characteristics of the animals.

	Dietary chitosan, ppm					
	0	300	600	1200	SEM	*P*-value
Carcass characteristics						
Carcass weight (kg)	89.39^b^	89.58^b^	88.66^b^	85.31^a^	0.986	0.011
Kill-out proportion (%)	76.31	77.23	77.28	77.70	0.385	0.095
Back fat depth (mm)	12.57	12.34	13.29	11.79	0.641	0.432
Carcass fat content (kg)	34.93^b^	34.63^ab^	34.86^b^	32.91^a^	0.749	0.046
Lean (%)	58.51	58.32	57.62	58.99	0.657	0.527
Lean yield (kg)	51.75	52.44	51.33	50.01	0.871	0.253
Loin eye muscledepth* (mm)	55.04	55.00	53.81	52.46	0.939	0.185
Carcass average dailygain (kg/day)	0.886^b^	0.898^b^	0.893^b^	0.816^a^	0.0166	0.002

Values represents least square means (n = 20).

Means with the same superscript within rows are not significantly different (P>0.05).

* Loin eye muscle depth is measured between the 10^th^ and 11^th^ ribs on pig carcasses and is used in the estimation of carcass leaness.

### Microbiology

The supplementation of chitosan had no effect on lactobacilli and *Enterobacteriaceae* populations (*P*>0.05) in the colon (data not shown).

### Serum Adipokines

**Figure 1:**
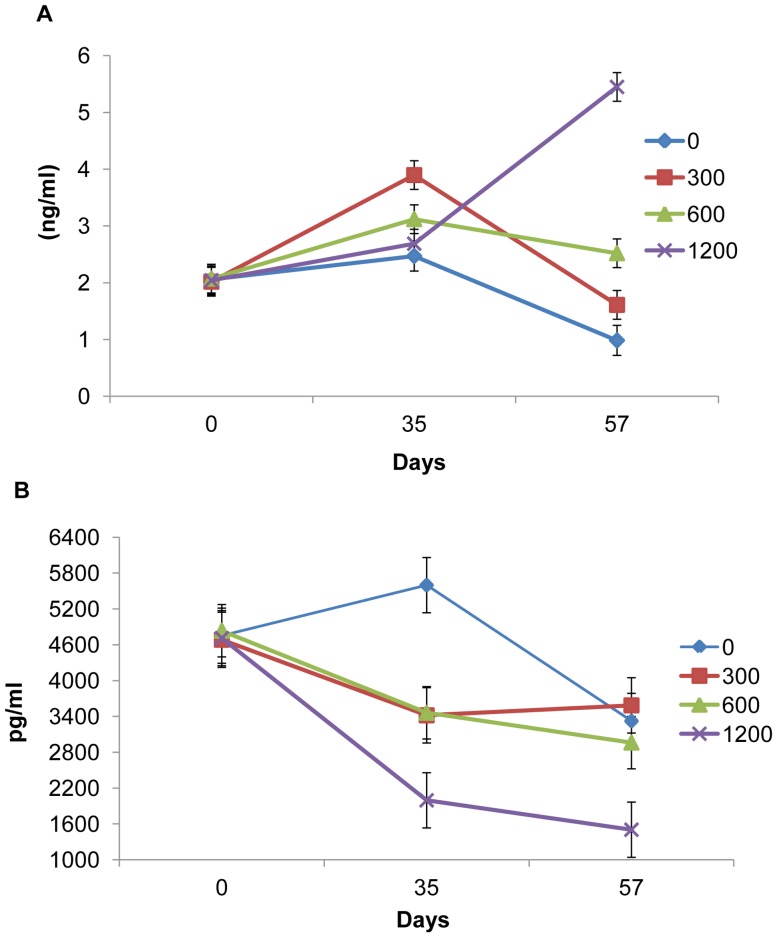
A. Differences in serum leptin concentration over time at days 0 (basal), 35 and 57**.** Values are mean with the SEM represented by vertical bars. **B.** Differences in serum C-reactive protein (CRP) concentration over time at days 0 (basal), 35 and 57. Values are mean with the SEM represented by vertical bars.

The effect of dietary treatment on serum leptin and CRP concentrations is presented in Figure 1. There was an interaction between dietary treatment and time of sampling on serum leptin concentrations (*P*<0.001). There was no effect of chitosan on serum leptin concentrations on day 0 and 35. However, animals offered 1200 ppm chitosan had a higher concentration of leptin when compared with the control (*P*<0.001) and other treatment groups (*P*<0.001) on day 57. There was an interaction between dietary treatment and time of sampling on serum CRP concentrations (*P*<0.05). There was no effect of the chitosan on serum CRP concentrations on day 0 and 35. However, animals offered 1200 ppm chitosan had a lower concentration of CRP when compared with the control (*P*<0.05) and the 300 ppm chitosan (*P*<0.05) groups on day 57. The majority of the IL-6 samples in the present study were below a minimal detectable level of IL-6 in serum, hence, were not included in the analysis.

### Relationships

There was a negative relationship between dietary feed intake and serum leptin concentrations (R^2^ = 0.591, *P*<0.001). There was a positive relationship between chitosan intake and serum leptin concentrations (R^2^ = 0.436, *P*<0.01). There was a negative relationship between chitosan intake and dietary feed intake (R^2^ = 0.410, *P*<0.05).

## Discussion

There is increasing interest in the use of natural resources as protective agents against obesity because of synthetic compounds having some harmful side-effects [28]. In the present study, supplementation of the 1200 ppm chitosan resulted in a significant decrease in body weight gain, which was associated with a decreased carcass weight and carcass fat content. In addition to chitosan increasing faecal fat excretion, the present study also showed that 1200 ppm chitosan decreased dietary intake, which was associated with increased serum leptin concentrations and a decrease in CRP concentrations. Hence, the results of this study highlight novel nutritional endocrine mechanisms by which chitosan exhibits anti-obesity properties *in vivo*.

Chitosan supplementation at 1200 ppm decreased crude fat digestibility in this experiment, suggesting that dietary supplementation of chitosan inhibited the intestinal absorption of dietary fat. This observation is supported by a number of previous studies in the literature. A number of *in vitro* studies have demonstrated that chitosan can bind fats and bile acids [29]. Although the mechanism is not fully understood, it has been suggested that chitosan dissolves in the stomach, emulsifying fat and forming a gel, which binds with the fat in the intestine, therefore interfering with the absorption of fat in the intestine [30], [31]. This insoluble complex then passes undigested through the large intestine and is naturally excreted. It has been proposed in other studies that the decreased body weight gain achieved by chitosan supplementation is related to its fat binding properties [9], [10]. Some research has shown that the supplementation of chitosan can bind to minerals and decrease mineral absorption in rats [9], [32]. Interestingly, the inclusion of chitosan had no effect on ash digestibility in the current study, which implies that chitosan was not binding to minerals.

In the current study, a decreased body weight gain was observed in the 1200 ppm chitosan treatment. The mechanism by which chitosan triggers body weight loss seems to be the result of increased leptin concentrations, given our observation of lower dietary intake with the supplementation of 1200 ppm chitosan in animals. These observed effects were rate dependent and only significant at an inclusion level that equates to 0.027 g per kg body weight. This would equate to a daily intake of chitosan of ∼2 g for an individual of 75 kg bodyweight. This intake should be a feasible intake for an adult human. This is an interesting observation as leptin is known to have a role in appetite suppression. Leptin is an important signal providing information about energy stores in the fat mass to the central nervous system and circulating leptin concentrations are correlated with the extent of obesity [33]. The negative relationship between dietary intake of the animals and serum leptin concentrations (R^2^ = 0.591) support this view. The hexosamine biosynthetic pathway has been proposed as a mechanism through which cells ‘sense’ the influx of nutrients, particularly glucose [34]. It has been suggested that the hexosamine pathway and its final product UDP-N-acetylglucosamine mediate leptin’s response to glucose. Leptin production in human adipose tissue was found to be regulated by the hexasamine pathway [35]. It has been reported that exposure of human adipose tissue to glucosamine increased leptin release in the culture medium [35]. Although the bioavailability of chitosan after oral administration to animals has not been well investigated, substantial amounts have been shown to be digested in the gut of poultry and rabbits [36], [37]. Indeed, chitinolytic enzymes that digest chitosan to glucosamine are present in the intestinal mucosa of mammals [6]. In the present experiment, the 1200 ppm chitosan, comprising copolymers of glucosamine, may have induced an up-regulation in serum leptin production in the later period of the study, which may be the underlying cause behind the dramatic decline in dietary intake and subsequent body weight loss.

Obesity is associated with both increased local adipose and more generalized systemic inflammation [38], [39]. The acute phase CRP is a sensitive marker of inflammation [40]. In the current study, a decreased body weight gain was observed in the 1200 ppm chitosan treatment during the later stages of the current study (day 35 to 57), which was associated with a significant decrease in serum CRP concentrations. In agreement with these observations, Heilbronn *et al.* [40] reported that energy restriction and weight loss in obese individuals have been associated with a decrease in plasma CRP concentrations. The precise anti-inflammatory mechanism of chitosan in decreasing serum CRP concentrations is not fully understood. The synthesis of CRP by the liver is largely regulated by the cytokine IL-6, and to a lesser extent, by other cytokines [41]. Furthermore, adipose tissue has been identified as an additional source of CRP [13]. Thus, a decrease in serum IL-6 concentrations may partly account for the decreasein serum CRP concentration achieved in the current study [42], [43]. Unfortunately, the majority of the IL-6 samples in the present study were below a minimum detectable level of IL-6 in the serum. Based on the substantial decrease in CRP serum concentrations, it would be beneficial to carry out IL-6 expression in liver samples in future experiments.

In general, gut microbial communities are considered an important factor affecting energy homeostasis. Among the many areas in obesity research, several studies revealed a close relationship between gut microbiota, nutrient utilization and energy storage by the host [44]. In the current study, there was no effect of chitosan supplementation on the number of *Enterobacteriaceae* or lactobacilli in the colon of the present study. This overall lack of an effect on these selected microflora populations, similar to the lack of effect on energy and protein digestibility, implies that the presence of chitosan in the diet did not elicit any deleterious effects on either of these two parameters.

As a depression in body weight gain was experienced only during the later 3 weeks of the experimental period (99–114 kg body weight), it is probable to suggest live-weight and fat deposition of the animals is a contributing factor associated with chitosan’s anti-obesity mode of action. The similarities in diet intake and body weight gain between treatments for the first 35 days (64–99 kg body weight) suggest that the animals needed to reach a certain mature body weight and fat deposition for supplementation of chitosan to have significant effect on body weight gain. It has been shown in other studies that each 10 kg increment from 100 kg body weight of pigs leads to reducing lean deposition and fat deposition increases dramatically in the later stages of the production period [45], [46]. Pigs start to accumulate body fat beginning at 45 kg bodyweight and that fat content increases disproportionally between 45 and 110 kg bodyweights [47]. Hence, there seems to be a body weight/adipocyte development stage effect involved in the anti-obesity mode of action of chitosan.

The present study has highlighted a novel endocrine mode of action for chitosan, with decreased body weight and dietary intake associated with increased serum leptin concentrations and decreased serum CRP concentrations. While the health promoting effects of chitosan was known to involve a decrease in lipid absorption at the level of the gut, our results suggest a more complex mode of action including the modulation of adipokines *in vivo*. The ability of chitosan to attenuate body weight loss at an endocrine level suggests it may be a promising agent in achieving a healthy body weight.
